# Poly[(μ_2_-4,4′-bipyridine-κ^2^ *N*:*N*′)bis­(μ_2_-2-phen­oxy­propionato-κ^2^ *O*:*O*′)cobalt(II)]

**DOI:** 10.1107/S1600536811042875

**Published:** 2011-10-22

**Authors:** Jin-Bei Shen, Jia-Lu Liu, Guo-Liang Zhao

**Affiliations:** a College of Chemistry and Life Sciences, Zhejiang Normal University, Jinhua 321004, Zhejiang, People’s Republic of China; b Zhejiang Normal University Xingzhi College, Jinhua, Zhejiang 321004, People’s Republic of China

## Abstract

In the polymeric title compound, [Co(C_9_H_9_O_3_)_2_(C_10_H_8_N_2_)]_*n*_, the Co^II^ ion is located on a twofold rotation axis and is six-coordinated by two N atoms from two 4,4′-bipyridine (4,4′-bipy) ligands in axial positions and by four O atoms from four 2-phen­oxy­propionate (POPA) anions in equatorial positions, defining a slightly distorted octa­hedral geometry. The carboxyl­ate group of the POPA anion displays a bis-monodentate mode, linking pairs of Co^II^ ions into a chain parallel to [001]. Adjacent chains are connected in a perpendicular manner through 4,4′-bipy ligands into layers parallel to (100). The 4,4′-bipy ligand is likewise located on a twofold rotation axis, with a dihedral angle between the two pyridine rings of 57.05 (7)°. C—H⋯O hydrogen-bonding inter­actions are present within the layers. π–π stacking inter­actions between the POPA benzene rings of neighbouring layers [centroid-to-centroid distance = 3.976 (3) Å and plane-to-plane distance = 3.618 (3) Å] stabilize the packing of the structure.

## Related literature

For background to phen­oxy­alkanoic acids, see: Müller & Buser (1997 ▶). For other metal complexes derived from phen­oxy­propionic acid, see: Shen *et al.* (2011*a* ▶,*b* ▶,*c* ▶,*d* ▶). For a related cobalt complex, see: Zhuang *et al.* (2007 ▶).
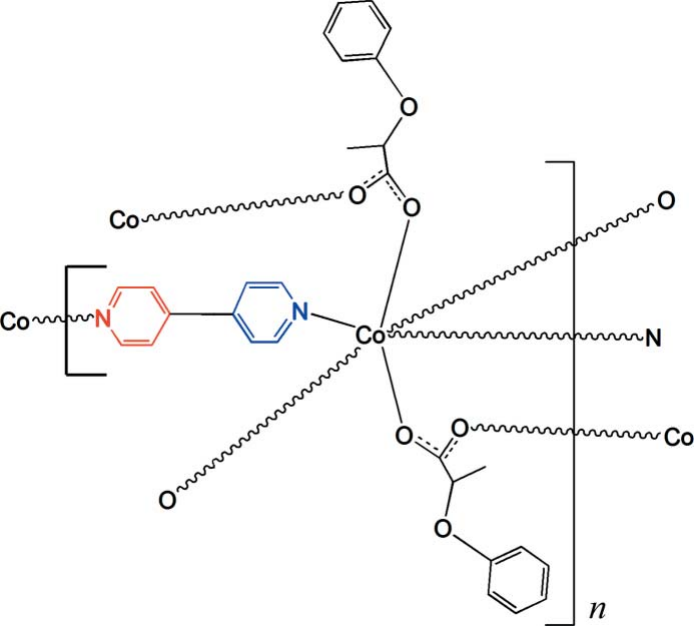


## Experimental

### 

#### Crystal data

[Co(C_9_H_9_O_3_)_2_(C_10_H_8_N_2_)]
*M* *_r_* = 545.44
Monoclinic, 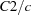

*a* = 23.6748 (14) Å
*b* = 11.6289 (7) Å
*c* = 9.6440 (6) Å
β = 96.353 (4)°
*V* = 2638.8 (3) Å^3^
*Z* = 4
Mo *K*α radiation
μ = 0.70 mm^−1^
*T* = 296 K
0.41 × 0.20 × 0.19 mm

#### Data collection

Bruker APEXII CCD diffractometer
Absorption correction: multi-scan (*SADABS*; Sheldrick, 1996 ▶) *T*_min_ = 0.847, *T*_max_ = 0.879
8421 measured reflections
2319 independent reflections
2053 reflections with *I* > 2σ(*I*)
*R*_int_ = 0.023

#### Refinement

*R*[*F* ^2^ > 2σ(*F* ^2^)] = 0.027
*wR*(*F* ^2^) = 0.068
*S* = 1.06
2319 reflections
170 parameters
H-atom parameters constrained
Δρ_max_ = 0.21 e Å^−3^
Δρ_min_ = −0.19 e Å^−3^

### 

Data collection: *APEX2* (Bruker, 2006 ▶); cell refinement: *SAINT* (Bruker, 2006 ▶); data reduction: *SAINT*; program(s) used to solve structure: *SHELXS97* (Sheldrick, 2008 ▶); program(s) used to refine structure: *SHELXL97* (Sheldrick, 2008 ▶); molecular graphics: *XP* in *SHELXTL* (Sheldrick, 2008 ▶) and *DIAMOND* (Brandenburg, 2006 ▶); software used to prepare material for publication: *publCIF* (Westrip, 2010 ▶).

## Supplementary Material

Crystal structure: contains datablock(s) I, global. DOI: 10.1107/S1600536811042875/wm2537sup1.cif

Structure factors: contains datablock(s) I. DOI: 10.1107/S1600536811042875/wm2537Isup2.hkl

Additional supplementary materials:  crystallographic information; 3D view; checkCIF report

## Figures and Tables

**Table 1:** Selected bond lengths (Å)

Co1—O3	2.0357 (12)
Co1—O2^i^	2.1275 (11)
Co1—N1	2.1989 (19)
Co1—N2	2.2051 (19)

Symmetry code: (i) 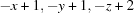.

**Table 2:** Hydrogen-bond geometry (Å, °)

*D*—H⋯*A*	*D*—H	H⋯*A*	*D*⋯*A*	*D*—H⋯*A*
C10—H10⋯O2^i^	0.93	2.51	3.079 (2)	120
C15—H15*A*⋯O3^i^	0.93	2.38	3.272 (2)	159

Symmetry code: (i) 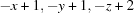.

**Fig. 1.**
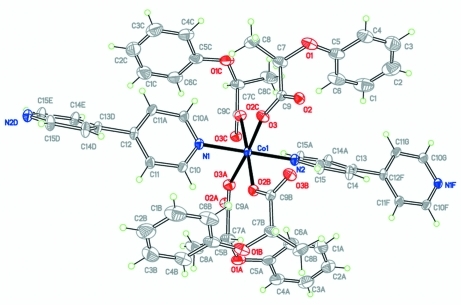
The molecular structure of the title complex, showing the atom-labelling scheme. Displacement ellipsoids are drawn at the 30% probability level. The atoms labelled with the suffix A, B, C, D, E, F, G are related by the symmetry operations (-x + 1, y, -z + 1.5), (-x + 1, -y + 1, -z + 2), (x, -y + 1, z - 1/2), (x, y + 1, z), (-x + 1, 1 + y, -z + 1.5), (x, y - 1, z), (-x + 1, y - 1, -z + 1.5), respectively.

**Fig. 2.**
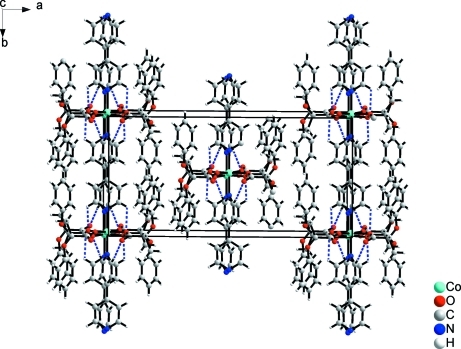
The layered arrangement of title compound, showing intralayer C—H···O interactions.

## supplementary crystallographic information

### Comment

The group of phenoxyalkanoic acids include important herbicides. The desired biological activity is largely dependent on the length of the carbon chain of the alkanoic acid, the nature of the phenoxy group, and the position of its attachment to the carbon chain (Müller & Buser, 1997). Therefore the structures of metal complexes of 2-phenoxypropionic acid became interesting for us. Recently, we have reported some results in this regard (Shen *et al.*, 2011*a*,*b*,*c*,*d*). Here, we describe a new Co^II^ complex with 4,4'-bipyridine (4,4'-bipy) as a co-ligand.

The structure of the polymeric title complex is shown in Fig. 1. The Co^II^ ion is located on a twofold rotation axis and is six-coordinated by four carboxylate O atoms from four POPA ligands and two N atoms of two 4,4'-bipy ligands in an octahedral geometry. The Co—O distances are 2.0357 (12) and 2.1275 (11) Å, and the Co—N distances are 2.1989 (19) and 2.2051 (19) Å, all of which are similar to related structures (Zhuang *et al.*, 2007). The 4,4'-bipy ligand exhibits symmetry 2, with a dihedral angle between the two pyridine rings of 57.05 (7)°. The carboxylate groups of the POPA anions display a bis-monodentate mode, bridging pairs of Co^II^ ions into chains parallel to [001]. The 4,4'-bipy molecules connect these chains perpendicularly, resulting in a layered arrangement parallel to (100) (Fig. 2).

As also shown in Fig. 2, intra-layer C—H···O hydrogen bonds between the C atoms of 4,4'-bipy ligands and carboxylate O atoms are present. Adjacent layers are stacked along [100] through π—π interactions between benzene rings of the POPA anions, with centroid–centroid and plane to plane distance of 3.976 (3) Å and 3.618 (3) Å, respectively.

### Experimental

Reagents and solvents used were of commercially available quality and were not further purified before using. 2-Phenoxyproionic acid (0.332 g, 2 mmol), 4,4'-bipy (0.156 g, 1 mmol) were mixed in distilled water (30 ml), NaOH (1 *M*) was added dropwise to the solution to adjust a pH of 5–6, then CoCl_2_.6H_2_O (0.238 g, 1 mmol) was added and the mixture sealed in a 50 ml stainless steel reactor and kept at 433 K for 3 d. The reactor was then cooled to room temperature at a speed of 5 K h^-1^. Red single crystals were obtained in high yield and filtered off from the solution.

### Refinement

The H atoms bonded to C and N atoms were positioned geometrically and refined using a riding model [aliphatic C—H = 0.96 Å (*U*_iso_(H) = 1.5*U*_eq_(C)), aromatic C—H = 0.93 Å (*U*_iso_(H) = 1.2*U*_eq_(C))].

### Crystal data

**Table:**

[Co(C_9_H_9_O_3_)_2_(C_10_H_8_N_2_)]	*F*(000) = 1132
*M**_r_* = 545.44	*D*_x_ = 1.373 Mg m^−^^3^
Monoclinic, *C*2/*c*	Mo *K*α radiation, λ = 0.71073 Å
Hall symbol: -C 2yc	Cell parameters from 3897 reflections
*a* = 23.6748 (14) Å	θ = 1.7–25.0°
*b* = 11.6289 (7) Å	µ = 0.70 mm^−^^1^
*c* = 9.6440 (6) Å	*T* = 296 K
β = 96.353 (4)°	Block, red
*V* = 2638.8 (3) Å^3^	0.41 × 0.20 × 0.19 mm
*Z* = 4	

### Data collection

**Table:**

Bruker APEXII CCD diffractometer	2319 independent reflections
Radiation source: fine-focus sealed tube	2053 reflections with *I* > 2σ(*I*)
graphite	*R*_int_ = 0.023
φ and ω scans	θ_max_ = 25.0°, θ_min_ = 1.7°
Absorption correction: multi-scan (*SADABS*; Sheldrick, 1996)	*h* = −28→26
*T*_min_ = 0.847, *T*_max_ = 0.879	*k* = −13→11
8421 measured reflections	*l* = −11→11

### Refinement

**Table:**

Refinement on *F*^2^	Primary atom site location: structure-invariant direct methods
Least-squares matrix: full	Secondary atom site location: difference Fourier map
*R*[*F*^2^ > 2σ(*F*^2^)] = 0.027	Hydrogen site location: inferred from neighbouring sites
*wR*(*F*^2^) = 0.068	H-atom parameters constrained
*S* = 1.06	*w* = 1/[σ^2^(*F*_o_^2^) + (0.0338*P*)^2^ + 1.1007*P*] where *P* = (*F*_o_^2^ + 2*F*_c_^2^)/3
2319 reflections	(Δ/σ)_max_ < 0.001
170 parameters	Δρ_max_ = 0.21 e Å^−^^3^
0 restraints	Δρ_min_ = −0.19 e Å^−^^3^

### Special details

**Table:**

Geometry. All e.s.d.'s (except the e.s.d. in the dihedral angle between two l.s. planes) are estimated using the full covariance matrix. The cell e.s.d.'s are taken into account individually in the estimation of e.s.d.'s in distances, angles and torsion angles; correlations between e.s.d.'s in cell parameters are only used when they are defined by crystal symmetry. An approximate (isotropic) treatment of cell e.s.d.'s is used for estimating e.s.d.'s involving l.s. planes.
Refinement. Refinement of *F*^2^ against ALL reflections. The weighted *R*-factor *wR* and goodness of fit *S* are based on *F*^2^, conventional *R*-factors *R* are based on *F*, with *F* set to zero for negative *F*^2^. The threshold expression of *F*^2^ > σ(*F*^2^) is used only for calculating *R*-factors(gt) *etc*. and is not relevant to the choice of reflections for refinement. *R*-factors based on *F*^2^ are statistically about twice as large as those based on *F*, and *R*-factors based on ALL data will be even larger.

### Fractional atomic coordinates and isotropic or equivalent isotropic displacement parameters (Å^2^)

**Table:**

	*x*	*y*	*z*	*U*_iso_*/*U*_eq_	
Co1	0.5000	0.52263 (3)	0.7500	0.02374 (11)	
O3	0.55380 (5)	0.53098 (11)	0.92961 (11)	0.0393 (3)	
C9	0.58754 (7)	0.49847 (13)	1.02991 (16)	0.0281 (4)	
N1	0.5000	0.71171 (16)	0.7500	0.0290 (4)	
C8	0.67426 (10)	0.6225 (2)	1.0358 (3)	0.0794 (8)	
H8A	0.7136	0.6252	1.0199	0.119*	
H8B	0.6708	0.6423	1.1312	0.119*	
H8C	0.6530	0.6762	0.9751	0.119*	
C7	0.65102 (8)	0.50059 (19)	1.0060 (2)	0.0493 (5)	
H7	0.6548	0.4805	0.9088	0.059*	
C11	0.45124 (8)	0.89365 (15)	0.76775 (19)	0.0384 (4)	
H11	0.4177	0.9315	0.7812	0.046*	
O1	0.68453 (5)	0.42260 (14)	1.09706 (14)	0.0555 (4)	
C10	0.45282 (7)	0.77274 (15)	0.76537 (19)	0.0367 (4)	
H10	0.4194	0.7328	0.7749	0.044*	
C6	0.64660 (10)	0.2551 (2)	0.9605 (3)	0.0699 (7)	
H6	0.6246	0.3010	0.8965	0.084*	
C1	0.64664 (11)	0.1349 (3)	0.9439 (3)	0.0883 (9)	
H1	0.6251	0.1027	0.8671	0.106*	
C5	0.67937 (8)	0.3048 (2)	1.0721 (2)	0.0536 (6)	
C4	0.71055 (10)	0.2322 (2)	1.1675 (2)	0.0651 (7)	
H4	0.7324	0.2641	1.2440	0.078*	
C3	0.70968 (11)	0.1124 (3)	1.1507 (3)	0.0810 (8)	
H3	0.7308	0.0661	1.2158	0.097*	
C2	0.67765 (12)	0.0629 (3)	1.0379 (4)	0.0906 (9)	
H2	0.6770	−0.0164	1.0256	0.109*	
C12	0.5000	0.95685 (19)	0.7500	0.0307 (5)	
O2	0.57552 (5)	0.47394 (10)	1.14927 (11)	0.0339 (3)	
C13	0.5000	0.08758 (19)	0.7500	0.0294 (5)	
N2	0.5000	0.33300 (16)	0.7500	0.0311 (4)	
C14	0.48321 (8)	0.15074 (14)	0.86106 (17)	0.0390 (4)	
H14A	0.4708	0.1130	0.9371	0.047*	
C15	0.48508 (8)	0.27175 (15)	0.85836 (17)	0.0380 (4)	
H15A	0.4754	0.3115	0.9360	0.046*	

### Atomic displacement parameters (Å^2^)

**Table:**

	*U*^11^	*U*^22^	*U*^33^	*U*^12^	*U*^13^	*U*^23^
Co1	0.03125 (19)	0.01885 (18)	0.02070 (16)	0.000	0.00108 (12)	0.000
O3	0.0410 (7)	0.0518 (8)	0.0235 (6)	0.0044 (6)	−0.0037 (5)	−0.0006 (5)
C9	0.0328 (9)	0.0268 (10)	0.0250 (8)	−0.0021 (7)	0.0040 (7)	−0.0023 (6)
N1	0.0354 (11)	0.0228 (11)	0.0283 (10)	0.000	0.0020 (8)	0.000
C8	0.0486 (14)	0.088 (2)	0.1002 (19)	−0.0260 (13)	0.0020 (13)	0.0290 (16)
C7	0.0350 (11)	0.0761 (16)	0.0378 (10)	0.0012 (10)	0.0077 (8)	0.0059 (10)
C11	0.0383 (10)	0.0266 (10)	0.0519 (10)	0.0060 (8)	0.0123 (8)	0.0033 (8)
O1	0.0366 (8)	0.0757 (11)	0.0525 (8)	0.0119 (7)	−0.0024 (6)	−0.0041 (8)
C10	0.0350 (10)	0.0263 (10)	0.0497 (10)	−0.0025 (8)	0.0088 (8)	0.0017 (8)
C6	0.0475 (14)	0.096 (2)	0.0644 (15)	0.0133 (13)	0.0001 (11)	−0.0174 (14)
C1	0.0584 (17)	0.102 (2)	0.103 (2)	0.0051 (16)	0.0024 (15)	−0.0423 (19)
C5	0.0315 (10)	0.0771 (17)	0.0535 (12)	0.0076 (10)	0.0104 (9)	−0.0093 (11)
C4	0.0461 (13)	0.086 (2)	0.0623 (14)	0.0115 (12)	0.0026 (11)	−0.0031 (13)
C3	0.0592 (16)	0.084 (2)	0.101 (2)	0.0153 (15)	0.0129 (15)	0.0090 (17)
C2	0.0567 (17)	0.079 (2)	0.139 (3)	0.0018 (16)	0.0263 (18)	−0.024 (2)
C12	0.0462 (15)	0.0207 (14)	0.0255 (11)	0.000	0.0046 (10)	0.000
O2	0.0351 (7)	0.0398 (7)	0.0276 (6)	0.0029 (5)	0.0070 (5)	0.0071 (5)
C13	0.0388 (14)	0.0197 (13)	0.0294 (11)	0.000	0.0022 (10)	0.000
N2	0.0419 (12)	0.0229 (11)	0.0282 (10)	0.000	0.0019 (9)	0.000
C14	0.0627 (12)	0.0250 (10)	0.0312 (9)	0.0026 (9)	0.0133 (8)	0.0049 (7)
C15	0.0605 (12)	0.0258 (10)	0.0292 (8)	0.0051 (9)	0.0119 (8)	−0.0018 (7)

### Geometric parameters (Å, °)

**Table:**

Co1—O3^i^	2.0357 (12)	C6—C5	1.382 (3)
Co1—O3	2.0357 (12)	C6—C1	1.407 (4)
Co1—O2^ii^	2.1275 (11)	C6—H6	0.9300
Co1—O2^iii^	2.1275 (11)	C1—C2	1.384 (4)
Co1—N1	2.1989 (19)	C1—H1	0.9300
Co1—N2	2.2051 (19)	C5—C4	1.398 (3)
O3—C9	1.243 (2)	C4—C3	1.402 (4)
C9—O2	1.2489 (18)	C4—H4	0.9300
C9—C7	1.546 (2)	C3—C2	1.381 (4)
N1—C10	1.3453 (19)	C3—H3	0.9300
N1—C10^i^	1.3453 (19)	C2—H2	0.9300
C8—C7	1.537 (3)	C12—C11^i^	1.395 (2)
C8—H8A	0.9600	C12—C13^iv^	1.520 (3)
C8—H8B	0.9600	O2—Co1^iii^	2.1275 (11)
C8—H8C	0.9600	C13—C14	1.3924 (19)
C7—O1	1.438 (2)	C13—C14^i^	1.392 (2)
C7—H7	0.9800	C13—C12^v^	1.520 (3)
C11—C12	1.395 (2)	N2—C15^i^	1.3439 (19)
C11—C10	1.407 (2)	N2—C15	1.3439 (19)
C11—H11	0.9300	C14—C15	1.408 (2)
O1—C5	1.394 (3)	C14—H14A	0.9300
C10—H10	0.9300	C15—H15A	0.9300
			
O3^i^—Co1—O3	174.53 (7)	N1—C10—C11	123.57 (16)
O3^i^—Co1—O2^ii^	95.10 (5)	N1—C10—H10	118.2
O3—Co1—O2^ii^	84.80 (5)	C11—C10—H10	118.2
O3^i^—Co1—O2^iii^	84.80 (5)	C5—C6—C1	119.8 (3)
O3—Co1—O2^iii^	95.10 (5)	C5—C6—H6	120.1
O2^ii^—Co1—O2^iii^	177.85 (6)	C1—C6—H6	120.1
O3^i^—Co1—N1	87.26 (4)	C2—C1—C6	122.3 (3)
O3—Co1—N1	87.26 (4)	C2—C1—H1	118.9
O2^ii^—Co1—N1	88.93 (3)	C6—C1—H1	118.9
O2^iii^—Co1—N1	88.93 (3)	C6—C5—O1	125.2 (2)
O3^i^—Co1—N2	92.74 (4)	C6—C5—C4	118.1 (2)
O3—Co1—N2	92.74 (4)	O1—C5—C4	116.7 (2)
O2^ii^—Co1—N2	91.07 (3)	C5—C4—C3	121.6 (2)
O2^iii^—Co1—N2	91.07 (3)	C5—C4—H4	119.2
N1—Co1—N2	180.0	C3—C4—H4	119.2
C9—O3—Co1	159.26 (12)	C2—C3—C4	120.4 (3)
O3—C9—O2	126.46 (16)	C2—C3—H3	119.8
O3—C9—C7	115.51 (14)	C4—C3—H3	119.8
O2—C9—C7	117.75 (15)	C3—C2—C1	118.0 (3)
C10—N1—C10^i^	116.3 (2)	C3—C2—H2	121.0
C10—N1—Co1	121.84 (10)	C1—C2—H2	121.0
C10^i^—N1—Co1	121.84 (10)	C11—C12—C11^i^	116.4 (2)
C7—C8—H8A	109.5	C11—C12—C13^iv^	121.80 (10)
C7—C8—H8B	109.5	C11^i^—C12—C13^iv^	121.80 (10)
H8A—C8—H8B	109.5	C9—O2—Co1^iii^	134.85 (11)
C7—C8—H8C	109.5	C14—C13—C14^i^	116.3 (2)
H8A—C8—H8C	109.5	C14—C13—C12^v^	121.84 (10)
H8B—C8—H8C	109.5	C14^i^—C13—C12^v^	121.84 (10)
O1—C7—C8	107.80 (18)	C15^i^—N2—C15	116.0 (2)
O1—C7—C9	112.24 (15)	C15^i^—N2—Co1	122.01 (10)
C8—C7—C9	108.70 (17)	C15—N2—Co1	122.01 (10)
O1—C7—H7	109.4	C13—C14—C15	120.04 (15)
C8—C7—H7	109.4	C13—C14—H14A	120.0
C9—C7—H7	109.4	C15—C14—H14A	120.0
C12—C11—C10	120.05 (16)	N2—C15—C14	123.75 (15)
C12—C11—H11	120.0	N2—C15—H15A	118.1
C10—C11—H11	120.0	C14—C15—H15A	118.1
C5—O1—C7	118.84 (16)		
			
O2^ii^—Co1—O3—C9	−81.2 (3)	C1—C6—C5—C4	−1.7 (3)
O2^iii^—Co1—O3—C9	101.0 (3)	C7—O1—C5—C6	4.0 (3)
N1—Co1—O3—C9	−170.3 (3)	C7—O1—C5—C4	−176.79 (16)
N2—Co1—O3—C9	9.7 (3)	C6—C5—C4—C3	0.9 (3)
Co1—O3—C9—O2	−96.6 (3)	O1—C5—C4—C3	−178.37 (19)
Co1—O3—C9—C7	89.7 (3)	C5—C4—C3—C2	0.1 (4)
O3^i^—Co1—N1—C10	65.25 (10)	C4—C3—C2—C1	−0.3 (4)
O3—Co1—N1—C10	−114.75 (10)	C6—C1—C2—C3	−0.4 (4)
O2^ii^—Co1—N1—C10	160.41 (10)	C10—C11—C12—C11^i^	−0.79 (12)
O2^iii^—Co1—N1—C10	−19.59 (10)	C10—C11—C12—C13^iv^	179.21 (12)
O3^i^—Co1—N1—C10^i^	−114.75 (10)	O3—C9—O2—Co1^iii^	−2.7 (3)
O3—Co1—N1—C10^i^	65.25 (10)	C7—C9—O2—Co1^iii^	170.92 (12)
O2^ii^—Co1—N1—C10^i^	−19.59 (10)	O3^i^—Co1—N2—C15^i^	56.44 (11)
O2^iii^—Co1—N1—C10^i^	160.41 (10)	O3—Co1—N2—C15^i^	−123.56 (11)
O3—C9—C7—O1	−156.70 (16)	O2^ii^—Co1—N2—C15^i^	−38.72 (10)
O2—C9—C7—O1	29.0 (2)	O2^iii^—Co1—N2—C15^i^	141.28 (10)
O3—C9—C7—C8	84.2 (2)	O3^i^—Co1—N2—C15	−123.56 (11)
O2—C9—C7—C8	−90.2 (2)	O3—Co1—N2—C15	56.44 (11)
C8—C7—O1—C5	−167.58 (16)	O2^ii^—Co1—N2—C15	141.28 (11)
C9—C7—O1—C5	72.7 (2)	O2^iii^—Co1—N2—C15	−38.72 (11)
C10^i^—N1—C10—C11	−0.85 (13)	C14^i^—C13—C14—C15	−1.47 (13)
Co1—N1—C10—C11	179.15 (13)	C12^v^—C13—C14—C15	178.53 (13)
C12—C11—C10—N1	1.7 (3)	C15^i^—N2—C15—C14	−1.59 (14)
C5—C6—C1—C2	1.5 (4)	Co1—N2—C15—C14	178.41 (14)
C1—C6—C5—O1	177.6 (2)	C13—C14—C15—N2	3.2 (3)

Symmetry codes: (i) −*x*+1, *y*, −*z*+3/2; (ii) *x*, −*y*+1, *z*−1/2; (iii) −*x*+1, −*y*+1, −*z*+2; (iv) *x*, *y*+1, *z*; (v) *x*, *y*−1, *z*.

### Hydrogen-bond geometry (Å, °)

**Table:**

*D*—H···*A*	*D*—H	H···*A*	*D*···*A*	*D*—H···*A*
C10—H10···O2^iii^	0.93	2.51	3.079 (2)	120.
C15—H15A···O3^iii^	0.93	2.38	3.272 (2)	159.

Symmetry codes: (iii) −*x*+1, −*y*+1, −*z*+2.
